# On the feature specificity of value-driven attention

**DOI:** 10.1371/journal.pone.0177491

**Published:** 2017-05-09

**Authors:** Brian A. Anderson

**Affiliations:** Texas A&M University, College Station, Texas, United States of America; University of Verona, ITALY

## Abstract

When an otherwise inconspicuous stimulus is learned to predict a reward, this stimulus will automatically capture visual attention. This learned attentional bias is not specific to the precise object previously associated with reward, but can be observed for different stimuli that share a defining feature with the reward cue. Under certain circumstances, value-driven attentional biases can even transfer to new contexts in which the reward cues were not previously experienced, and can also be evident for different exemplars of a stimulus category, suggesting some degree of tolerance in the scope of the underlying bias. Whether a match to a reward-predictive feature is necessary to support value-driven attention, or whether similar-looking features also receive some degree of elevated priority following associative reward learning, remains an open question. Here, I examine the impact of learned associations between reward and red- and green-colored stimuli on the processing of other colors. The findings show that even though other colors experienced during training were non-predictive with respect to reward, the speed with which targets possessing these colors were identified in a subsequent test phase was affected by their similarity to the high-value color. Thus, value-driven attentional biases for stimulus features are imprecise, as would be predicted by a sensory gain model of value-driven attention.

## Introduction

In order to survive and thrive, it is important that organisms pay close attention to the stimuli in their environment that signal the potential availability of reward [1]. Although reward incentives can modulate goal-directed attention mechanisms [2–8], reward can also have a more direct impact on the attention system [9–12]. Specifically, when an object has been learned to predict high reward, this object will capture the attention of an observer even when it is completely task-irrelevant and no longer rewarded, suggesting an automatic value-driven attentional bias [see ref. 13 for a recent review].

Few studies have explored the specificity of the learning underlying value-driven attention. Attentional biases for stimuli possessing a feature previously associated with high value can be observed even when the task context and specific object possessing the feature are different from those experienced during learning [14]. Value-driven attentional biases can also be observed for a range of exemplars from a category that reliably predicts high reward [15–17], although the presence of common features across exemplars of a category was not explicitly controlled or manipulated in these studies.

Under certain conditions, in contrast, value-driven attentional biases can also be narrowly tuned. When contextual information is diagnostic with regards to whether a particular stimulus feature will be followed by reward, value-driven attentional biases for that feature have been shown to be specific to the rewarded context [18,19]. To the degree that the trained stimulus representation underlying value-driven attention is largely on the level of an object category, as is possible given its neural correlates in the ventral visual stream [15,20–23], perceptually distinguishable non-matching features might fail to capture attention. However, if the trained stimulus representation underlying value-driven attention encompasses, at least in some part, shifts in the gain of feature-specific responses in early visual processing [13,24–29], it might be predicted that features that share some representational overlap with the previously reward-predictive feature would also experience some degree of bias. This prediction arises out of the representational nature of features such as color and orientation in early visual processing, which reflect population responses from neurons that exhibit tuning curves [30,31].

The effect of value learning for one stimulus feature on the processing of different stimulus features has never been explicitly examined. In the present study, I provide a test of whether learning to associate a particular feature with high value affects the processing of stimuli possessing a range of other features along that same dimension. Specifically, I measured the speed with which a color target was identified based on its similarity to a color that was predictive of high value in a prior training phase. In the traditional version of the value-driven attentional capture paradigm introduced by Anderson and colleagues [9,32,33], each of two target colors predicts a different amount of reward for accurately identifying the target. Six different non-target colors also appear during the reward training, which are entirely non-predictive with respect to reward. Collapsing across three different prior experiments using the same design, I classified the shape-defined target during the subsequent unrewarded test phase with respect to whether its color was more similar to the previously high-value color from training, the previously low-value color from training, or neutral with respect to the trained colors. By combining across multiple experiments, more subtle effects that any one experiment would be underpowered to detect can be examined [34].

## Materials and methods

The present study pooled data from three different experiments in which participants performed the exact same task. One study was Experiment 3 of the original demonstration of value-driven attention [9], the second was the healthy control group for a study of depressed college students using this task [32], and the third was from a replication study of the original demonstration [33].

### Participants

A total of 84 participants were included in the present dataset, 24 from [9], 20 from [32], and 40 from [33]. Data from 10 participants from ref. 32 were not available because detailed information concerning the color of each stimulus was not recorded for these participants. All were recruited from the Johns Hopkins University community, all reported normal or corrected-to-normal visual acuity and normal color vision, and all were between the ages of 18 and 35 inclusive. All participants provided written informed consent, and all of the study procedures were approved by the Johns Hopkins University Institutional Review Board and conformed to the principles outlined in the Declaration of Helsinki [35].

### Training phase

#### Stimuli

**Fig 1:**
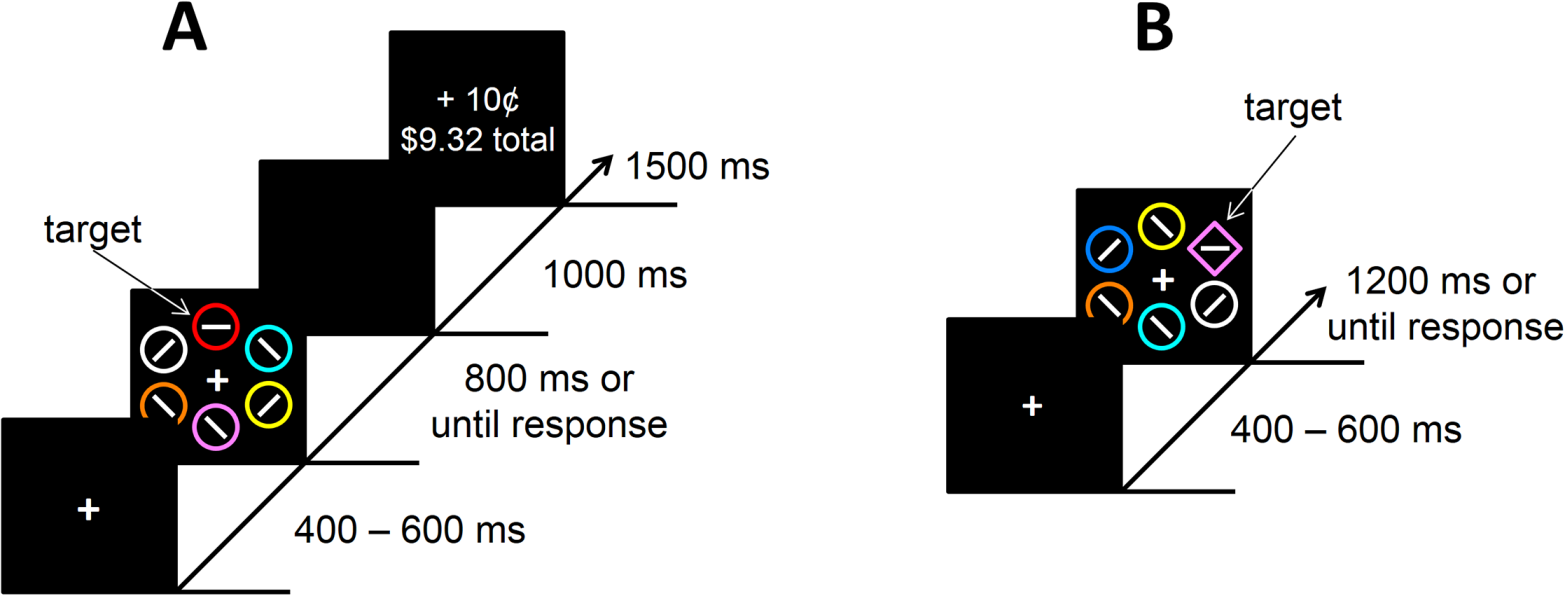
Experimental task. (A) Training phase. Participants searched for a target defined by its color (red or green) and reported the orientation of the bar within the target. Each correct response earned a monetary reward, which tended to be larger for one target color than for the other. (B) Test phase. Participants now searched for a shape-defined target (diamond among circles or circle among diamonds). The color of the shapes was explicitly task-irrelevant. The color of the target could vary in its similar to the high-value color experienced during training.

Each trial consisted of a fixation display, a search array, and a feedback display (see Fig 1A). The fixation display contained a white fixation cross (.5° x .5° visual angle) presented in the center of the screen against a black background, and the search array consisted of the fixation cross surrounded by six colored circles (each 2.3° x 2.3°) placed at equal intervals on an imaginary circle with a radius of 5°. The target was defined as the red [CIEL*A*B*: 53 80 67] or green [CIEL*A*B*: 88–86 83] circle, exactly one of which was presented on each trial; the color of each non-target circle was drawn from the set {blue [CIEL*A*B*: 55 19–71], cyan [CIEL*A*B*: 91–48–14], pink [CIEL*A*B*: 72 65–42], orange [CIEL*A*B*: 67 43 74], yellow [CIEL*A*B*: 97–22 94], white [CIEL*A*B*: 100 0 0]} without replacement. Inside the target circle, a white bar was oriented either vertically or horizontally, and inside each of the non-targets, a white bar was tilted at 45° to the left or to the right (randomly determined for each non-target). The feedback display indicated the amount of monetary reward earned on the current trial, as well as the total accumulated reward. Note that the monitors used in these studies were not gamma-corrected, and the CIEL*A*B* coordinates were determined assuming typical monitor performance for the type of monitor used. Thus, these values should be considered estimates.

#### Design

One of the two color targets (counterbalanced across participants) was followed by a high reward of 10¢ on 80% of correct trials and a low reward of 2¢ on the remaining 20% (high-value target); for the other color target, these percentages were reversed (low-value target). Each color target appeared in each location equally-often, and trials were presented in a random order. The five non-target colors used on each trial were randomly selected from the six available non-target colors, such that each non-target color was equally familiar and not differently predictive of any task variable (accordingly, there was no significant difference in the number of times a non-target color from each of the three categories used in data analysis was experienced during training: *F* < 1).

#### Procedure

The training phase consisted of 240 trials, which were preceded by 50 practice trials. Each trial began with the presentation of the fixation display for a randomly varying interval of 400, 500, or 600 ms. The search array then appeared and remained on screen until a response was made or 800 ms had elapsed, after which the trial timed out. The search array was followed by a blank screen for 1000 ms, the reward feedback display for 1500 ms, and a 1000 ms inter-trial interval (ITI).

Participants made a forced-choice target identification by pressing the "z" and the "m" keys on a standard US-layout keyboard for the vertically- and horizontally-orientated bars within the targets, respectively. Correct responses were followed by monetary reward feedback in which a small amount of money was added to the participant's total earnings. Incorrect responses or responses that were too slow were followed by feedback indicating 0¢ had been earned. If the trial timed out, the computer emitted a 500 ms 1000 Hz tone.

### Test phase

#### Stimuli

Each trial consisted of a fixation display, a search array, and a feedback display (see Fig 1B). The six shapes now consisted of either a diamond among circles or a circle among diamonds, and the target was defined as the unique shape. On a subset of the trials, one of the non-target shapes was rendered in the color of a formerly reward-associated target from the training phase (referred to as the *valuable distractor*); the target was never red or green. The feedback display only informed participants if their prior response was correct or not.

#### Design

Target identity, target location, distractor identity, and distractor location were fully crossed and counterbalanced, and trials were presented in a random order. Valuable distractors were presented on 50% of the trials, half of which were high-value distractors and half of which were low-value distractors (high- and low-value color from the training phase, respectively).

#### Procedure

The training phase and test phase were separated by a short break of approximately 5 min. In the test phase, participants were instructed to ignore the color of the shapes and to focus on identifying the unique shape using the same orientation-to-response mapping. The test phase consisted of 240 trials, which were preceded by 20 practice (distractor absent) trials. The search array was followed immediately by non-reward feedback for 1000 ms in the event of an incorrect response (this display was omitted following a correct response in ref. 32 and 33, but not in ref. 9 in which correct feedback was also provided) and then by a 500 ms ITI; no monetary rewards were given. Trials timed out after 1200 ms. As in the training phase, if the trial timed out, the computer emitted a 500 ms 1000 Hz tone. Upon completion of the experiment, participants were paid the cumulative reward they had earned in the training phase.

### Data analysis

Only correct responses were included in the mean response time (RT) for each participant, and RTs exceeding three standard deviations of the mean for each condition for each participant were trimmed. Analysis focused on distractor absent trials to avoid any effects of the distractor color influencing the perception of other simultaneously-presented colors. The data were broken down by whether the target color was more similar to the high-value color from training, more similar to the low-value color from training, or neutral with respect to red and green. Neutral colors were defined as blue and white, orange and pink as more similar to red, and cyan and yellow as more similar to green, which is consistent with their relative positions in CIEL*A*B* space (averaging across the two colors defined as more similar to red and the two more similar to green, when comparing to red targets, ΔE_00_ [36] was 31.99 for red-similar colors and 80.71 for green-similar colors, while when comparing to green targets, ΔE_00_ was 28.75 for green-similar colors and 67.79 for red-similar colors). Note that, because only six non-trained colors were used for six stimulus positions, this assignment equally partitioned all distractor-absent trials: whenever a color was not the target color, it was necessarily a non-target (distractor) color on that trial.

Mean RT and accuracy for each condition for each participant were computed using custom Matlab scripts, and group analyses were performed using SPSS software. Cohen's d was computed as a measure of effect size by dividing the mean difference score by the standard deviation of the difference scores in Matlab. Holm-Bonferroni correction was applied to the alpha criterion for each of the two planned pairwise comparisons reported in the Results when determining significance. Overall performance did not differ based on which of the three prior studies [9,32,33] participants were drawn from, nor did this factor interact with target color condition, *F*s < 0.4, *p*s > .81, so further analyses collapsed across study.

## Results

**Fig 2:**
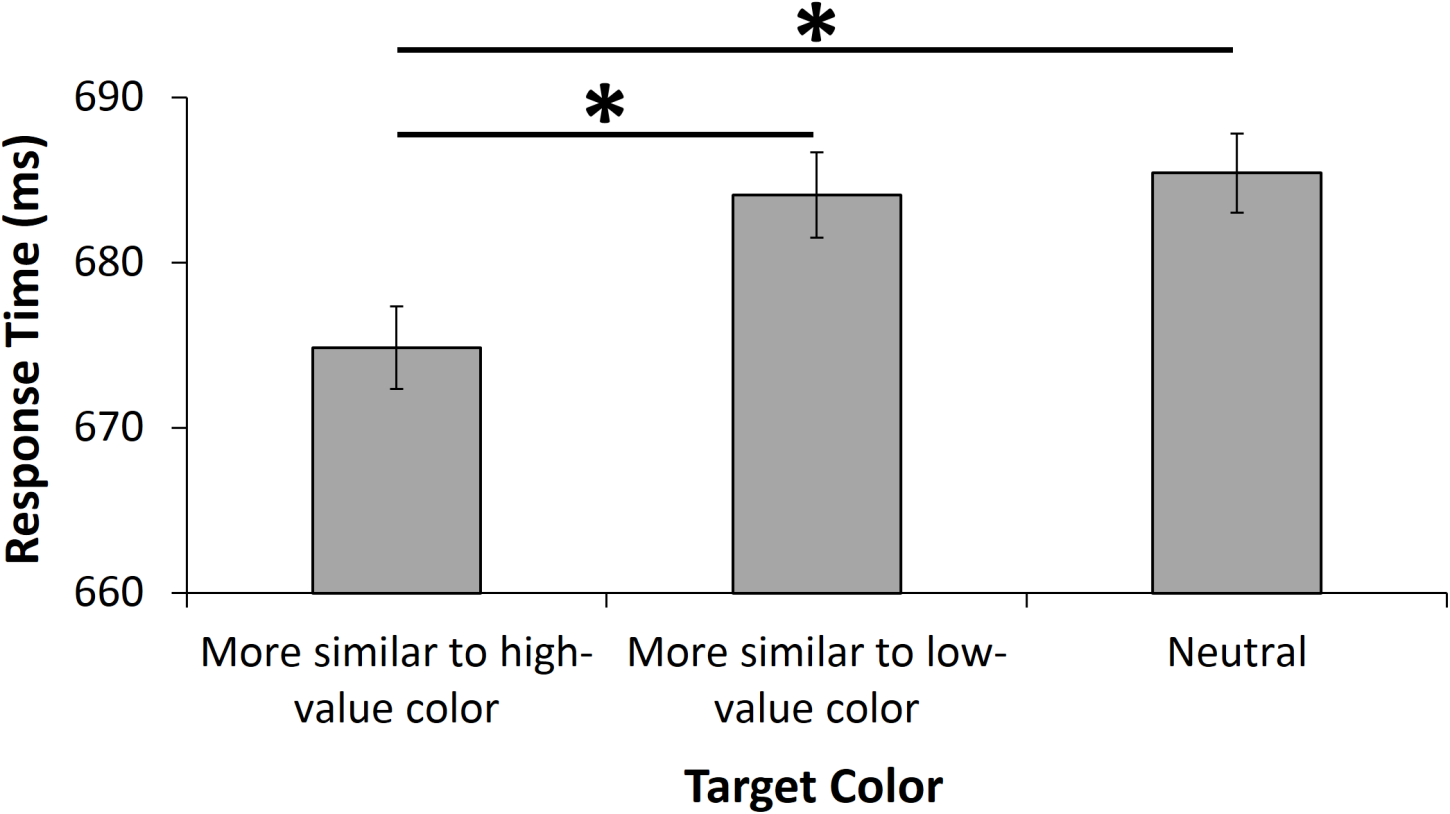
Behavioral data. Response time by target color condition during the test phase. Error bars reflect within-subjects confidence intervals *p < .05.

Mean RT for distractor-absent trials in the test phase when the target color was: (1) more similar to the high-value color from training, (2) more similar to the low-value color from training, and (3) neutral with respect to red and green was submitted to a one-way repeated measures analysis of variance. This analysis revealed a significant effect of target color condition, *F*(2,166) = 3.53, *p* = .031, η^2^_p_ = .041. Planned pairwise comparisons revealed that RT was significantly faster when the target color was more similar to the high-value color (mean = 675 ms, stdev = 71.8) than when it was more similar to the low-value color (mean = 684 ms, stdev = 72.0), *t*(83) = 2.05, *p* = .044, *d* = .22, and also significantly faster when the target color was more similar to the high-value color than when it was classified as a neutral color (mean = 685 ms, stdev = 71.3), *t*(83) = 2.50, *p* = .014, *d* = .27 (see Fig 2). In the case of the former, the assignment of color to condition was counterbalanced across participants such that each pair of colors was represented in each value condition equally often; thus, the difference in performance between these two conditions can only be explained in terms of prior value learning. Accuracy did not differ by target color condition, *F*(2,166) = 1.32, *p* = .270, and followed the same pattern as RT (high-value: 86.5%, low value: 85.6%, neutral: 85.2%), suggesting the absence of any speed-accuracy tradeoff. Condition means for each individual participant are available as supporting information (S1 File).

## Discussion

The findings provide support for the idea that value-driven attentional biases are not feature-specific and can "spill over" to stimuli possessing features that are similar to a feature previously associated with high reward. This is in spite of the fact that the similar features themselves were explicitly non-predictive of reward during learning. In this way, much like goal-contingent attentional capture [37], the feature-specificity of value-driven attention is distinctly imprecise [38–40].

The findings of the present study would be predicted by the hypothesis that the modulation of sensory gain in early visual areas plays a role in value-driven attention [13,24–29]. Specifically, if dopaminergic reward signals serve as teaching signals to early visual areas such as V1 [21,25,29,41–43], amplifying the gain of neurons whose feature-evoked responses predicted the reward, the consequences of this shift in sensitivity should not be restricted to the processing of the rewarded feature. This is because feature-selective neurons in early stages of visual processing exhibit a tuning curve, responding maximally to a preferred feature but also to similar features in a graded fashion [30,31]. Therefore, the enhanced response of these neurons to reward-predictive stimuli, as is evident, for example, in the P1 event-related potential component [24], should also be evident to varying degrees in response to a range of similar features that would activate overlapping cell populations.

On the other hand, it is important to note that the findings of the present study could also be explained by the modulation of stimulus representations in object-selective regions of the brain, which have also been shown to be sensitive to reward history [15,20–23]. Especially given the simple nature of the stimuli used in the present study (colored shapes appearing at fixed locations), these stimuli may activate partially overlapping representations in object-selective areas. To the extent that the degree of overlap is related to color similarity, the same principles as described above would apply here as well. It has been shown that the ability of reward history to modulate stimulus processing can occur at the level of an entire object category such as people and cars [15], suggesting some degree of tolerance to feature variety that could support the generalization of learning across a range of similar features.

In the present study, the colors experienced during the test phase were all non-rewarded colors experienced during training. As such, it is unclear to what degree the observed selection bias reflects enhanced priority for colors more similar to the high-value color or de-prioritization of colors less similar to the high-value color (as in, e.g., ref. 12). A related source of ambiguity in the present study concerns the spatial specificity of the observed selection bias. Stimuli possessing a feature that matches the feature learned to predict high reward capture spatial attention, which is evident in eye movements [44–46], cuing effects [47,48], and lateralized stimulus processing in the brain [20,21,23,49]. The selection bias observed in the present study may reflect this same sort of selection mechanism (or its inhibition for non-similar colors), or it could reflect a fundamentally different mechanism such as increased attentional dwell time [50] on non-targets more similar to the previously high-value feature or increased non-spatial filtering costs [51] caused by these stimuli. It would be informative for future studies to use features in the test phase that were never experienced during training, and to employ spatially-specific measures of stimulus selection such as eye movements.

Color similarity was not systematically manipulated in the present study, which leveraged relationships that were present in existing datasets. As such, the present study does not afford a systematic exploration of the nature of the relationship between reward learning, feature similarity, and attentional bias beyond category-level distinctions of feature relationships. Both color and luminance varied across the stimuli that were used; although raw differences in luminance cannot explain the results given the counterbalancing of value and color, luminance differences may have played a role in the stimulus representations that were ultimately modulated by reward learning (i.e., subsumed within the definition of "similarity").

On the surface, the present findings, as well as findings from studies investigating the relationship between feature similarity and goal-contingent attentional capture [38–40], conflict with those of one study showing surround-suppression effects when directing attention to color-defined stimuli [52]. At least under certain circumstances, attending to one color is associated with the inhibition of similar colors [52]. Although the reasons for this discrepancy are unclear, one major difference between ref. 52 and investigations of feature similarity in attentional capture paradigms (as in the present study and refs. 38–40) is that in ref. 52, the two similar colors were presented simultaneously. Thus, the inhibition of similar features observed in ref. 52 may reflect a goal-directed mechanism for resolving high levels of stimulus competition.

When a stimulus feature is learned to predict reward, the consequences of this learning on the attention system are broad and extend beyond the specific trained feature. The resulting bias supports generalization of learning to novel stimuli that bear some resemblance to that which was rewarded. A similar degree of non-specificity is evident in goal-contingent attentional capture [38–40], possibly reflecting a broader principle by which the attention system assigns priority in feature space.

## Supporting information

S1 File Condition means for individual participants. (XLSX)
